# Multivariate locally stationary 2D wavelet processes with application to colour texture analysis

**DOI:** 10.1007/s11222-016-9675-9

**Published:** 2016-07-01

**Authors:** Sarah L. Taylor, Idris A. Eckley, Matthew A. Nunes

**Affiliations:** Department of Mathematics and Statistics, Fylde College, Lancaster University, Lancaster, LA1 4YF UK

**Keywords:** Random field, Local spectrum, Local coherence, Colour texture, Wavelets

## Abstract

In this article we propose a novel framework for the modelling of non-stationary multivariate lattice processes. Our approach extends the locally stationary wavelet paradigm into the multivariate two-dimensional setting. As such the framework we develop permits the estimation of a spatially localised spectrum *within* a channel of interest and, more importantly, a localised cross-covariance which describes the localised coherence *between* channels. Associated estimation theory is also established which demonstrates that this multivariate spatial framework is properly defined and has suitable convergence properties. We also demonstrate how this model-based approach can be successfully used to classify a range of colour textures provided by an industrial collaborator, yielding superior results when compared against current state-of-the-art statistical image processing methods.

**Electronic supplementary material:**

The online version of this article (doi:10.1007/s11222-016-9675-9) contains supplementary material, which is available to authorized users.

## Introduction

Wavelet methods have enjoyed popularity for many years for statistical data analysis due to their ability to provide efficient representations of signals and images. For a recent overview of wavelet techniques, see for example Vidakovic (1999) or Nason (2008). Amongst many notable contributions, recently a significant body of work has emerged in the area locally stationary wavelet time series. This stems from the seminal work of Nason et al. (2000). Notable contributions include work on forecasting (Fryzlewicz et al. 2003), changepoint analysis (Killick et al. 2013), signal classification (Fryzlewicz and Ombao 2009) and alias detection (Eckley and Nason 2014).

In two dimensions, this analysis falls into a class of problems known as texture analysis. Historically much of the work in this area has been targeted towards the analysis of greyscale (i.e. univariate) textured images. In recent years various contributions have been made to this area by the statistical community. These have typically sought to develop *short-memory*, multiscale models of the spatial covariance structure within textured images, see for example Eckley et al. (2010) or Mondal and Percival (2012). Within such contributions model-based estimates are used to distinguish between subtly different texture types. The multiscale framework is particularly appealing in this setting since it is well-documented that the mammalian visual system operates in a such a manner, see for example Daugman (1990) or Field (1999). See Petrou and Sevilla (2006) for a comprehensive introduction to this field.

In this article we adopt a formal, model-based approach to the analysis of *multivariate* two-dimensional random fields. Specifically, we develop a modelling framework which permits a multiscale decomposition of the second-order structure of a colour image. The novelty of the approach is that our proposed model considers *both* (i) within-component spatial covariance, and (ii) the spatially-localised coherence *between* components of a multivariate process. This latter quantity provides a local, *multiscale* measure of the (linear) dependence between components, and thus enables the model to accommodate realistic spatial non-stationarity whilst also representing the inherent multiscale structure of texture. Such multiscale measures of dependence have already been shown to be beneficial in other contexts, see for example Park et al. (2014) or Sanderson et al. (2010). An associated estimation scheme for model quantities is also developed.

Within the image analysis setting, our model lends itself to the analysis of *colour* textured images. Such images are typically multivariate in nature, containing three *channels* of spatial data, for example red, green and blue (RGB) or hue, saturation, value (HSV). These channels may, or may not, exhibit cross-channel dependence. Of course, we are by no means the first to consider this problem. See for example, work in the image processing community by Van de Wouwer et al. (1999) and Sengur (2008) and references therein. Some methods either fail to describe *both* the multichannel (colour) and textural aspects of “colour texture”, whilst others do not represent its inherent multiscale nature. We hence use our model estimation scheme as the basis of an example application of our modelling framework: a colour texture classification method. Both simulated and industrially-motivated examples demonstrate that our approach is able to differentiate between multivariate spatial processes which exhibit subtly different textural and colour properties, outperforming state-of-the-art competitor methods.

The remainder of the article is organised as follows. In Sect. 2 we introduce the multivariate locally stationary 2D modelling framework and also define key measures which describe the second-order structure within such processes: the locally stationary wavelet cross- and auto-spectra and locally stationary wavelet coherence. The estimation theory for such processes is established in Sect. 3 together with a simulated example which demonstrates the potential of our spectral estimators to describe localised structure. Finally in Sect. 4 we consider the application of our approach to two related texture classification challenges introduced by an industrial collaborator.

## The multivariate 2D locally stationary wavelet model

The *locally stationary two-dimensional wavelet* (LS2W) model for spatial fields has been proposed as a formal framework to represent spatial inhomogeneity whilst also capturing the inherent multiscale structure of texture (Eckley et al. 2010). Loosely speaking, by incorporating the multiscale structure of (non-decimated) wavelets within a spatial context it is possible to describe a spatially evolving structure which allows both for stationary structure (when sufficiently localised) and spatially diverse structures (when looked at from a distance).

In this section we introduce the multivariate 2D locally stationary wavelet process model, which extends earlier work by Eckley et al. (2010) to the multivariate setting. We also introduce various scale-based measures of variation which describe the spectral and cross-spectral behaviour locally within non-stationary images.

### The locally stationary wavelet model of a multivariate spatial process

We start by considering an *m*-dimensional spatial process, which we denote $\mathbf {X}_{\mathbf {r;R}} = [X_{\mathbf {r,R}}^{(1)}; X_{\mathbf {r,R}}^{(2)}; \ldots ; X_{\mathbf {r,R}}^{(m)}]'$. Here each element is an individual channel (i.e. spatial plane) of the multivariate image. We seek to describe the cross-channel dependence. To this end we define the multivariate 2D locally stationary wavelet process (LS2Wmv) model as follows.

#### Definition 1

A *m*-variate 2D locally stationary wavelet process process, $\mathbf {X_{\mathbf {r;R}}}$, is defined to have the following form:

1 $$\begin{aligned} X_{\mathbf {r},\mathbf {R}}^{(1)}= & {} \sum _{\eta } \sum _{\mathbf {u}} W^{(1)}_{\eta }(\mathbf {u}/\mathbf {R}) \psi _{\eta ,\mathbf {u-r}} \xi ^{(1)}_{\eta ,\mathbf {u}} \nonumber \\ \vdots \quad&\qquad \vdots \nonumber \\ X_{\mathbf {r},\mathbf {R}}^{(m)}= & {} \sum _{\eta } \sum _{\mathbf {u}} W^{(m)}_{\eta }(\mathbf {u}/\mathbf {R}) \psi _{\eta ,\mathbf {u-r}} \xi ^{(m)}_{\eta ,\mathbf {u}}. \end{aligned}$$

Here $\mathbf {r} = (r,s) \in \{0, \ldots , R-1\} \times \{0, \ldots , S-1\} = \mathcal {R}$ and $R=2^{k}$, $S=2^{n}$ for some $k, n \in \mathbb {N}$. The $\{W_{\eta }^{(i)}(\mathbf {u}/\mathbf {R})\}$ can be thought of as scale-direction-location dependent transfer functions in rescaled space, where the index *i* represents a particular channel of the multivariate image. The random variables $\{\xi ^{(i)}_{\eta ,\mathbf {u}}\}$ for $\mathbf {u} \in \mathcal {R}$ are a collection of zero-mean random orthonormal increments which encapsulate the cross-channel dependence of the process through Eq. (3) (see below) and $\{\psi _{\eta ,\mathbf {u}}\}$ are two-dimensional discrete non-decimated wavelets as defined in Eckley et al. (2010).

Note that each individual channel can be seen as a (univariate) LS2W process on a regular lattice. Note also that in the above, we adopt a simplified notation for a scale-direction pair. Instead of having two separate indices representing scale and direction (i.e. *j* and *l*), a combination of both provides a single index, $\eta$, each value of which represents a particular decomposition scale in a given direction. More specifically, we have $\eta :=\eta (j,l) = j + g(l)$ for all $j=1,\ldots ,J$ where $g(l)=0,J,2J$ and $l=v,h,d$.

#### Modelling assumptions for the LS2Wmv model

In order that a principled estimation theory for LS2Wmv processes can be developed, we require several modelling assumptions to control the degree of non-stationarity of the process and enable the identification of the local spectral structure. Specifically:

1. $\mathbb {E}[\xi ^{(i)}_{\eta ,\mathbf {u}}] = 0$ hence $\mathbb {E}(\mathbf {X}_{\mathbf {r}})=0$ for all $\eta$ and $\mathbf {u}$.
2. The increments of the process, $\{\xi ^{(i)}_{\eta ,\mathbf {u}}\}$, have the following properties
  2 $$\begin{aligned} \text{ Cov }[\xi _{\eta ,\mathbf {k}}^{i}, \xi _{\eta _{1},\varvec{m}}^{i}]= & {} \delta _{\eta ,\eta _{1}} \delta _{\mathbf {k},\varvec{m}} \end{aligned}$$
  3 $$\begin{aligned} \text{ Cov }[\xi _{\eta ,\mathbf {k}}^{p}, \xi _{\eta _{1},\varvec{m}}^{q}]= & {} \delta _{\eta ,\eta _{1}} \delta _{\mathbf {k},\varvec{m}} \rho ^{p,q}_{\eta }(\mathbf {k}/\mathbf {R}), \end{aligned}$$
  where $\delta _{\eta ,\eta _{1}}$ is the Kronecker delta, and as a consequence of extending to a multivariate setting, $\rho ^{p,q}_{\eta }(\mathbf {u}/\mathbf {R})$ represents the possible coherence structure between each pair (*p*, *q*) of image channels, where we assume the images are in-phase. This quantity is the *LS2W coherence* between channels *p* and *q*, which we discuss in more detail in the next section.
3. The LS2Wmv coherence in (3) is assumed to be Lipschitz continuous for each scale-direction pair with Lipschitz constants $R^{(p,q)}_{\eta }$ satisfying
  $$\begin{aligned} \sum _{\eta } 2^{2j(\eta )}R^{(p,q)}_{\eta } < \infty . \end{aligned}$$
4. Additionally, for each scale-direction $\eta$, the transfer function $W_{\eta }^{(i)}(\mathbf {z})$, is Lipschitz continuous with constants $L^{(i)}_{\eta }$ which are uniformly bounded in $\eta$ such that:
  $$\begin{aligned} \sum _{\eta } 2^{2j(\eta )} L^{(i)}_{\eta } < \infty . \end{aligned}$$

### Measuring local power and cross-channel coherence in LS2Wmv processes

It is well-known that the spectral structure of a signal or image can be used to describe its second-order behaviour (see for example Priestley (1981); Broughton and Bryan (2011)). For the multivariate setting on which we focus in this article, this leads to the consideration of (i) spatially localised wavelet spectra which represent the structure within a single channel, and (ii) cross-spectra which capture the structure across channels.

#### Definition 2

Let $X_{\mathbf {r}}^{(p)}$ and $X_{\mathbf {r}}^{(q)}$ be two channels of a LS2Wmv process with amplitude functions $W_{\eta }^{(p)}(\mathbf {z})$ and $W_{\eta }^{(q)}(\mathbf {z})$ respectively. Then the local wavelet cross-spectrum (LWCS) of the two channels $X_{\mathbf {r}}^{(p)}$ and $X_{\mathbf {r}}^{(q)}$ is given by

4 $$\begin{aligned} S^{p,q}_{\eta }(\mathbf {z}) = W_{\eta }^{(p)}(\mathbf {z}) W_{\eta }^{(q)}(\mathbf {z}) \rho ^{p,q}_{\eta }(\mathbf {z}), \end{aligned}$$

for $\mathbf {z} \in (0,1)^{2}$ and scale-direction $\eta$.

For the case where $p=q$, Eq. (4) gives the auto-spectra (the local wavelet spectrum (LWS) for each channel) as given in Eckley et al. (2010). The LWS provides a measure of the local contribution to the variance of each channel. Conversely, in the case where $p\ne q$ the local wavelet cross-spectra quantify the cross-covariance between channels at a specific (rescaled) location $\mathbf {z}$ and scale-direction $\eta$. To quantify the dependence between channels we first introduce the local cross-covariance (LCCV) and consider its relationship to the local cross-spectrum.

#### Definition 3

Let $c^{(p,q)}(\mathbf {z},\varvec{\tau })$ denote the local cross-covariance between channels *p* and *q* from a LS2Wmv process at lag $\varvec{\tau } \in \mathbb {Z}^{2}$. We define this function in terms of the local cross-spectrum by

5 $$\begin{aligned} c^{(p,q)}(\mathbf {z},\varvec{\tau }) = \sum _{\eta } S^{p,q}_{\eta }(\mathbf {z}) \varPsi _{\eta }(\varvec{\tau }), \end{aligned}$$

for $\varvec{\tau } \in \mathbb {Z}^{2}$, where $\varPsi _{\eta }(\varvec{\tau })= \sum _{\mathbf {v} \in \mathbb {Z}^2} \psi _{\eta ,\mathbf {v}}(\mathbf {0}) \psi _{\eta ,\mathbf {v}}(\mathbf {\varvec{\tau }})$ are the two-dimensional autocorrelation wavelets as defined in Eckley and Nason (2005).

It can be shown that given a choice of wavelet, this cross-spectral representation is unique. Moreover, it can be established that the LS2Wmv process cross-covariance, given by $c_{\mathbf {R}}^{(p,q)}(\mathbf {z},\varvec{\tau }) = \text{ Cov }\left( X_{[\mathbf {zR}]}^{(p)},X_{[\mathbf {zR}+\varvec{\tau }]}^{(q)}\right)$, asymptotically tends to local cross-covariance of each pair of channels.

#### Theorem 1

The LWCS for each *p* and *q* is uniquely defined given the corresponding LS2Wmv process. Moreover let $c^{(p,q)}(\mathbf {z},\varvec{\tau })$ denote the local cross-covariance for two channels *p* and *q* of an LS2Wmv process from Definition 3. Then

$$\begin{aligned} \left| c_{\mathbf {R}}^{(p,q)}(\mathbf {z},\varvec{\tau }) - c^{(p,q)}(\mathbf {z},\varvec{\tau })\right| = O\left( \frac{1}{\min \{R,S\}}\right) \end{aligned}$$

for $\varvec{\tau } \in \mathbb {Z}^{2}$ and $\mathbf {z} \in (0,1)^2$ as $R, S \rightarrow \infty$.

The proof of this result can be found in the appendix.

The LS2Wmv process quantity $\rho ^{p,q}_{\eta }(\mathbf {z})$ in Definition 2, which we term the *LS2Wmv coherence* between two channels *p* and *q*, is a direct measure of the linear dependence between the innovation sequences of two channels at scale-direction $\eta$. This measure can be defined as

6 $$\begin{aligned} \rho ^{p,q}_{\eta }(\mathbf {z}) = \frac{S^{p,q}_{\eta }(\mathbf {z})}{\sqrt{S_{\eta }^{(p)}(\mathbf {z}) S_{\eta }^{(q)}(\mathbf {z})}}, \end{aligned}$$

where the individual LWS of each channel, $S_{\eta }^{(p)}(\mathbf {z})$, together with the LWCS $S^{p,q}_{\eta }(\mathbf {z})$, provide a normalised measure of the relationship between two channels. The value of the coherence determines the level of dependence between two channels, with $+1$ and $-1$ indicating a positive and negative dependence respectively, and a value of zero showing no dependence at a given scale-direction $\eta$ and rescaled location $\mathbf {z}$.

In the next section we develop a rigorous estimation theory for the LS2Wmv process quantities introduced in this section, establishing desirable asymptotic properties of the estimators. These properties are analogous to those for univariate LS2W processes in Eckley et al. (2010) but consider the crucial cross-channel dependence in the LS2Wmv model.

## Estimation theory for LS2Wmv processes

Recall from standard Fourier theory that the traditional estimate of the spectrum is the square of the transformed process coefficients. In a similar fashion we define the (raw) cross-spectra as the product of empirical wavelet coefficients $d_{\eta ,\mathbf {u}}^{(i)}$ from individual channels with $d_{\eta ,\mathbf {u}}^{(i)} = \sum _{\mathbf {r}} X_{\mathbf {r}}^{(i)} \psi _{\eta , \mathbf {u-r}}$. Using these empirical coefficients we can define an estimator of the cross-spectrum of each pair of channels as the local (raw) wavelet cross-periodogram for two channels of a LS2Wmv process $X_{\mathbf {r}}^{(p)}$ and $X_{\mathbf {r}}^{(q)}$, given by $I_{\eta , \mathbf {u}}^{(p,q)} = d_{\eta ,\mathbf {u}}^{(p)}d_{\eta ,\mathbf {u}}^{(q)}.$

We now establish the statistical properties of the (raw) wavelet cross-periodogram as an estimator of the LWCS. In order to estimate the relationship between channels we use a pairwise approach. The proofs of the results of this section can be found in the appendix.

### Theorem 2

Let $X_{\mathbf {r}}^{(p)}$ and $X_{\mathbf {r}}^{(q)}$ be two channels of an LS2Wmv process. Then asymptotically, the expectation of the (raw) cross-periodogram between these two channels, $I_{\eta , \mathbf {s}}^{(p,q)}$, is given by

$$\begin{aligned} \mathbb {E}(I_{\eta , \mathbf {s}}^{(p,q)})&= \sum _{\eta _{1}} W_{\eta _{1}}^{(p)} \left( \frac{\mathbf {s}}{\mathbf {R}}\right) W_{\eta _{1}}^{(q)} \left( \frac{\mathbf {s}}{\mathbf {R}}\right) \rho ^{p,q}_{\eta _{1}} \left( \frac{\mathbf {s}}{\mathbf {R}}\right) A_{\eta _{1},\eta } \\&\quad + {\mathcal O}\left( \frac{1}{\min \{R,S\}}\right) \\&= \sum _{\eta _{1}} S^{p,q}_{\eta _{1}}\left( \frac{\mathbf {s}}{\mathbf {R}}\right) A_{\eta _{1},\eta } + {\mathcal O}\left( \frac{1}{\min \{R,S\}}\right) , \end{aligned}$$

where $\mathbf {R} = (R, S)$, with $R=2^k, S=2^n$ for some $k, n \in {\mathbb N}$. Similarly, the variance is given by

7 $$\begin{aligned} Var(I_{\eta , \mathbf {s}}^{(p,q)})= & {} \sum _{\eta _{1}} S_{\eta _{1}}^{(p)}\left( \frac{\mathbf {s}}{\mathbf {R}}\right) A_{\eta _{1},\eta } \sum _{\eta _{1}} S_{\eta _{1}}^{(q)}\left( \frac{\mathbf {s}}{\mathbf {R}}\right) A_{\eta _{1},\eta } \nonumber \\&+\, \bigg (\sum _{\eta _{1}}S^{p,q}_{\eta _{1}}\left( \frac{\mathbf {s}}{\mathbf {R}}\right) A_{\eta _{1},\eta }\bigg )^{2} + {\mathcal O}\left( \frac{2^{2j(\eta _{1})}}{\min \{R,S\}}\right) .\nonumber \\ \end{aligned}$$

Here $\eta$ is fixed and $j(\eta )$ simply refers to scale for each direction.

The theorem demonstrates that the local raw cross-periodogram is a biased estimator of the cross-spectrum. However, rather helpfully (7) indicates that an asymptotically unbiased estimator can be obtained by transforming the raw periodogram by $A^{-1}_{J}$, where $A_J$ denotes the (curtailed) two-dimensional discrete autocorrelation wavelet inner product matrix [see Eckley and Nason (2005) for more details, including results establishing the invertibility of $A_{J}$]. Theorem 2 also establishes that smoothing the raw periodogram estimator is required to achieve asymptotic consistency. We choose to use the Nadaraya-Watson kernel estimator (Nadaraya 1964; Watson 1964) for smoothing the cross-spectra. The estimator is given by the weighted average

8 $$\begin{aligned} \widetilde{I}^{(p,q)}_{\eta ,\mathbf {s}} = \sum _{\mathbf{u} \in \mathcal {R}} w_{\mathbf {u}} I^{(p,q)}_{\eta , \mathbf {u}}, \end{aligned}$$

where the lattice weights are given by $w_{\mathbf {u}}=\frac{K_{h}(||\mathbf {s-u}||}{\sum _{\mathbf {u}}K_{h}(||\mathbf {s-u}||)}$. In this expression $K_{h}(\cdot )$ is a (two-dimensional) kernel function with bandwidth *h*.

The asymptotic properties of the resulting smoothed cross-periodogram, $\tilde{I}$, are established in the following Theorem.

### Theorem 3

The (asymptotic) expectation of $\widetilde{I}^{(p,q)}_{\eta , \mathbf {s}}$ is given by,

9 $$\begin{aligned} \mathbb {E}(\widetilde{I}^{(p,q)}_{\eta , \mathbf {s}}) = \sum _{\eta _{1}} S^{p,q}_{\eta _{1}}\left( \mathbf {s/R}\right) A_{\eta _{1},\eta } + \frac{1}{2\lfloor h\rfloor +1} {\mathcal O} \left( \frac{1}{\min \{R,S\}}\right) . \end{aligned}$$

Furthermore, the variance of the smoothed cross-periodogram vanishes:

10 $$\begin{aligned} {\text {var}}(\widetilde{I}^{(p,q)}_{\eta ,\mathbf {s}}) \rightarrow 0, \end{aligned}$$

as $$h,R, S \rightarrow \infty $$ with $$(h/\min \{R,S\}) \rightarrow 0$$.

**Fig. 1:**
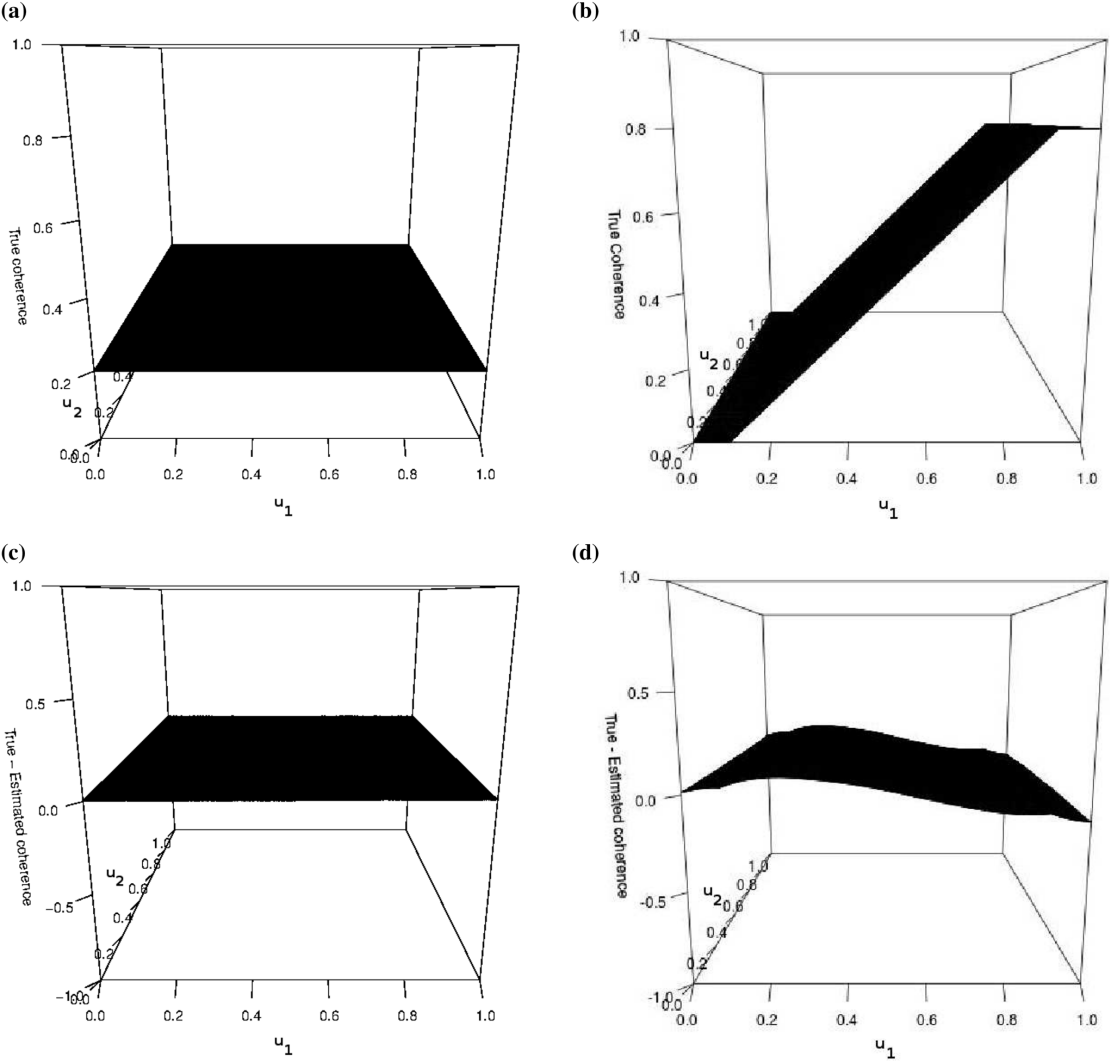
Plots of the coherence at the finest scale: **a** corresponds to the coherence between the first and second channel $\rho ^{1,2}_{(1,v)}(u_1,u_2)=\rho ^{1,2}_{(1,v)}(\mathbf {z})$, whereas **b** the coherence between the second and third channel, $\rho ^{2,3}_{(1,v)}(\mathbf {z})$; **c** shows the average estimation error at the finest scale between the first and second channel; **d** corresponds to the the average estimation error at the finest scale between the second and third channel

Defining the smoothed corrected cross-periodogram $\widehat{I}$ as

11 $$\begin{aligned} \widehat{I}^{(p,q)}_{\eta ,\mathbf {s}} = \sum _{\eta _{1}} A_{\eta ,\eta _{1}}^{-1} \widetilde{I}^{(p,q)}_{\eta _{1},\mathbf {s}}, \end{aligned}$$

by bias-correcting the spectrum estimate with $A^{-1}_J$ we obtain the result

12 $$\begin{aligned} \mathbb {E}(\widehat{I}_{\eta , \mathbf {s}}^{(p,q)}) = S^{p,q}_{\eta }(\mathbf {s/R}) + \frac{1}{2\lfloor h\rfloor +1} {\mathcal O}\left( \frac{1}{\min \{R,S\}}\right) . \end{aligned}$$

In other words, the estimator is asymptotically unbiased. This expression holds for any pair of channels *p* and *q* of the multivariate image. Taken together Theorems 2 and 3 establish the consistency of the estimator of the LWCS for each pair of channels. Note that this estimate, along with the estimate of the LWS, can be used in equation (6) to estimate the coherence.

### Example

In order to demonstrate our multivariate model and coherence measure, we now present an example of data simulated using a (square) trivariate LS2Wmv process with given spectral and coherence structure. The spectrum is constant at all scales and directions, i.e. $S^{1,1}_{\eta }(\mathbf {u/R}) = S^{2,2}_{\eta }(\mathbf {u/R}) = S^{3,3}_{\eta }(\mathbf {u/R}) =2$. Further, the coherence between the first and second channel is $\rho ^{p,q}_{\eta }(\mathbf {u/R})=0.2$ and the coherence between the second and third channel increases linearly along the horizontal axis between 0 and 0.8 as demonstrated in Fig. 1. The LS2Wmv process realisations in this and subsequent sections were generated using Gaussian increments.

We estimate this coherence using the methods described in Sect. 3. Note that results in this section as well as subsequent examples in this article were produced in the *R* statistical computing environment (R Core Team 2013), using modifications to the code from the *LS2W* package (Eckley and Nason 2011a, b). Fig. 1 shows the difference between the true and estimated coherence at the finest scale in the vertical direction based on averaging $K=100$ simulations. We observe similar results for the other directions. The estimate for the coherences between the first two channels is very good, signified by the low estimation error. We also obtain a reasonable estimate for the more difficult coherence specification between the second and third channel, the estimate capturing the general structure. Other simulated examples show a similar degree of estimation accuracy.

We now consider the application of our modelling approach to colour texture.

## Application of the LS2W model to colour image classification

In this section we apply our modelling framework to synthetic colour textures as well as images arising from an industrial application of product evaluation. We start by introducing a feature vector based on the LS2Wmv model (Sect. 2) which forms the basis of our colour image classification procedure. The key to our approach is finding the coherence between the colour channels as we believe the additional information provided by the coherence will allow more subtle differentiation between visually similar images. The feature vector we suggest considers the location average auto- and cross-spectral structure and the average local wavelet coherence at each scale-direction pair, since these measures encapsulate changes in the second-order (textural) structure of a colour image. Algorithm 1 describes the method which we use to obtain a feature vector for an LS2Wmv process with three channels.
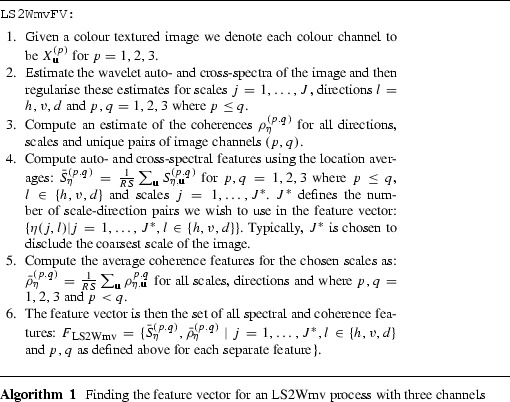


In order to show the full potential of our method we choose to compare it against three approaches. In particular, we compare our technique against the wavelet-based method of Van de Wouwer et al. (1999) as it is a popular approach using a fast algorithm which specifically focusses on cross-correlation measures. The alternative wavelet approach of Sengur (2008) is included in our study since the author reports better classification results than Van de Wouwer et al. (1999) for their chosen examples. Due to the ubiquity of Fourier methods in the literature we also compare against an alternative method based on the Fourier spectrum and Fourier coherence (Van Heel et al. 1982; Saxton and Baumeister 1982). From now on we shall refer to the approaches in Sengur (2008) as ‘Sengur’, Van de Wouwer et al. (1999) as ‘VdW’ and the Fourier approach as ‘Fourier’.

In what follows, we consider colour images with three channels corresponding to a RGB colour representation, but the method can equally be applied to other colour space specifications. For recent reviews of feature extraction and colour texture classification, see for example Prats-Montalbán et al. (2011).

**Fig. 2:**
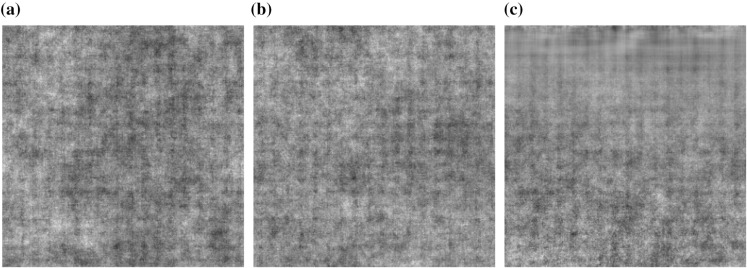
Examples of visually similar simulated colour textures for classification using the procedure in Sect. 4 generated with the LS2Wmv model

**Table 1:** Percentage of textures classified correctly using the different feature vectors described in the text for the textures displayed in Figs. 2, 3, 5 and 6

% Classified correctly	Method			
	Fourier	Sengur	VdW	LS2Wmv
Simulated textures (Fig. 2)	67.33	74	85.33	94.66
Simulated textures (Fig. 3)	26	76	61.33	84.66
Hair colourants (Fig. 5)	96	87.33	79.33	96.67
Hair preparations (Fig. 6)	77.33	68	90	91.33

### The classification procedure

To demonstrate the power of the LS2Wmv approach against the alternative methods described above, we classify a test set of images representing a number of colour texture images. To perform the classification experiment we use a nearest centroid classifier based on linear discriminant analysis (LDA), one of many potential approaches commonly used in practice which could be used to classify such data. See for example Hastie et al. (2001) or Parker (2010) for more details on this classification method. The classification experiment is as follows. We sample fifty sub-images of dimension *m* x *m* from the upper half of each image in the set. These sub-images comprise our training set. Another set of fifty sampled sub-images is used as test images to classify in order to assess the performance of each method. We perform LDA on the training set using the feature vector highlighted in Algorithm 1; for each test sub-image, the LDA-transformed feature vector is calculated, and the image is assigned a texture class as the class whose centroid is closest in Euclidean distance. This is then repeated for the competitor Fourier and wavelet classification feature vectors. We assess classification performance of each method accordingly using the average classification rate over the fifty test sub-images.

### Synthetic Examples

To illustrate the potential of the LS2Wmv feature vector described in Algorithm 1 in texture classification tasks, we simulate a number of colour textures of dimension $256 \times 256$ with different colour texture structure, and then use the classification procedure described above (Sect. 4) to classify 50 sampled sub-images of each texture, using another 50 sub-images as a training set.

For the first example, we use the LS2Wmv model (1) to simulate a number of colour textures for classification. The three LS2Wmv processes in the classification experiment are defined as follows. The first process has a spectrum which is constant at all scales and directions ($S^{1,1}_{\eta }(\mathbf {u/R}) = S^{2,2}_{\eta }(\mathbf {u/R}) = S^{3,3}_{\eta }(\mathbf {u/R}) =2$), with the coherence between the first and second channel being $\rho ^{1,2}_{\eta }(\mathbf {u/R})=0.2$; the coherence between the second and third channel is the same at all scales and directions and is set to 0.4. The second process is similar in structure to the first process, except we specify a non-stationary coherence between channels 2 and 3, namely it is set to 0.4 for half the pixels, and -0.4 for the other half, i.e. $\rho ^{2,3}_{\eta }((u_1,u_2)/R)=0.4$ for $u_1\in \{1,\dots ,128\}$ and $\rho ^{2,3}_{\eta }((u_1,u_2)/R)=-0.4$ for $u_1\in \{129,\dots ,256\}$. The third process has the same coherence structure as Process 2, but has a non-stationary spectral structure at all scales and directions. In particular, the spectral structure increases linearly from 0 to 0.8 as depicted in Fig. 1. Example realisations of the three processes can be seen in Fig. 2.

**Fig. 3:**
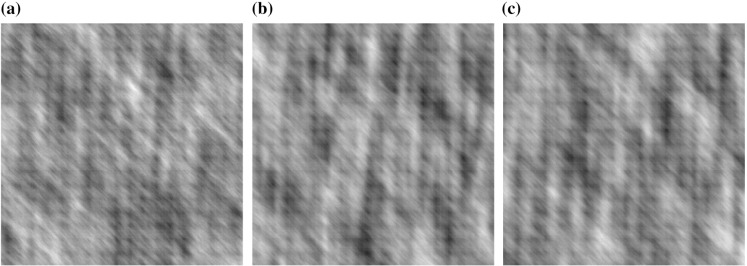
Examples of visually similar simulated colour textures for classification using the procedure in Sect. 4 generated with the model of coregionalization

The experiment attempts to classify the sub-images from the three processes. The resulting proportion of correctly classified images for the experiment is shown in Table 1. Whilst being difficult to distinguish visually, the LS2Wmv feature vector permits a substantially better classification of the processes due to being able to model the potential non-stationarity in both coherence and spectral structure (Table 1).

To examine the classification potential of our modelling framework further, we repeated the classification experiment using a second example of colour textures. Specifically, we simulate colour textures from a *linear model of coregionalization*. In this class of models, each channel of a colour texture is defined as a linear combination of independent univariate random fields [see e.g. Wackernagel (2003) or Gelfand et al. (2004)]. In other words,

$$\begin{aligned} X^{(p)}_{\mathbf {u}} = \sum _{j=1}^{k} m_{p,j} Y_{j,\mathbf {u}}, \quad p=1,2,3 \end{aligned}$$

for independent random fields $Y_{j,\mathbf {u}}$ defined at spatial locations $\mathbf {u}$. For the processes in this example, we specify that the $Y_{j}$ have stationary, anisotropic covariance functions $C_{\kappa , \mu , \phi }(\cdot )$ from the Gneiting (1999) class of models, as described in Schlather et al. (2015). In these functions, the parameters $\kappa$ and $\mu$ characterize the form and smoothness of the covariance, whilst $\phi$ is an angle representing the degree of the anisotropy. In particular, for the experiment we define three different processes

$$\begin{aligned} \mathbf {X}^{A}_{\mathbf {u}}= & {} \left( \begin{array}{cc} 0.6 &{} 0.6\\ 0.8 &{} 0.5\\ 0.7 &{} 0.8\\ \end{array}\right) \left( \begin{array}{c} Y^{A}_{1,\mathbf {u}}\\ Y^{A}_{2,\mathbf {u}} \end{array}\right) \\ \mathbf {X}^{B}_{\mathbf {u}}= & {} \left( \begin{array}{cc} 0.6 &{} 0.6\\ 0.5 &{} 0.8\\ 0.8 &{} 0.7\\ \end{array}\right) \left( \begin{array}{c} Y^{B}_{1,\mathbf {u}}\\ Y^{B}_{2,\mathbf {u}} \end{array}\right) \\ \mathbf {X}^{C}_{\mathbf {u}}= & {} \left( \begin{array}{cc} 0.6 &{} 0.6\\ 0.5 &{} 0.8\\ 0.8 &{} 0.7\\ \end{array}\right) \left( \begin{array}{c} Y^{C}_{1,\mathbf {u}}\\ Y^{C}_{2,\mathbf {u}} \end{array}\right) . \end{aligned}$$

Moreover, we specify that $Y^{A}_{1,\mathbf {u}}$, $Y^{B}_{1,\mathbf {u}}$ and $Y^{C}_{1,\mathbf {u}}$ all have Gneiting covariance functions $C_{0, 2, 0}(\cdot )$; $Y^{A}_{2,\mathbf {u}}$, $Y^{B}_{2,\mathbf {u}}$ and $Y^{C}_{2,\mathbf {u}}$ have covariances $C_{3, 2, \frac{\pi }{4}}(\cdot )$, $C_{2, 2, \frac{\pi }{4}}(\cdot )$ and $C_{2, 1, \frac{\pi }{4}}(\cdot )$ respectively. More explicit forms for these functions for particular values of $\kappa$ can be found in Schlather et al. (2015). See also Wendland (2004) for more details on these processes. The textures were simulated using the *RandomFields* R package (Schlather 2014). The textures used in the experiment can be seen in Fig. 3. Visually, these textures can be seen to exhibit vertical and diagonal structure, but it is difficult to distinguish between them.

Similar to the first example, the experiment attempts to classify the 50 sub-images from the three processes. The proportion of correctly classified sub-images is shown in Table 1. The Fourier method performs poorly in the experiment. The Sengur and VdW are an improvement; the LS2Wmv feature vector achieves good classification via its ability to model the differing structure in the processes, despite the similarities between the images.

### Application of the LS2W model to the classification of hair images

**Fig. 4:**
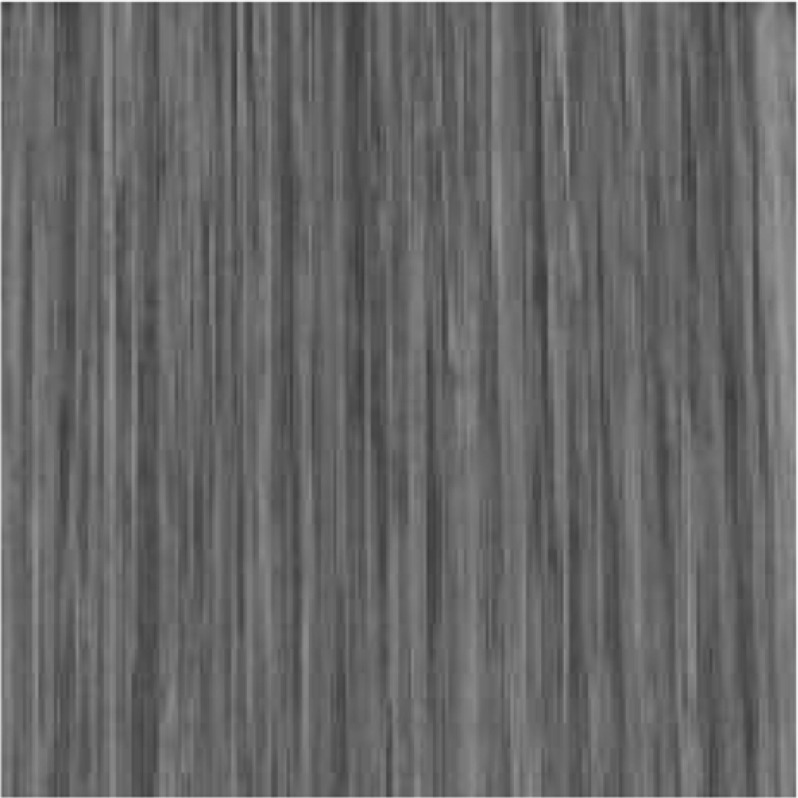
Typical hair sample analysed in industrial product evaluation

We now consider an application of our modelling framework to a colour texture analysis problem encountered by an industrial collaborator. In many industrial settings, it is of considerable interest to be able to discriminate between different hair images based on their textural properties, for example, to indicate the age of a product or its variability under different conditions. Until recently such images were analysed by experienced image analysts. However, such a task can be challenging even to the human eye, and as suggested by Liang et al. (2012), manual methods of image inspection are subjectively dependent on human vision. As such, a more principled approach for classifying such images is required. Thus it is of interest to see which of the four methods performs well in these differing cases. A typical swatch of hair analysed in the experiments is depicted in Fig. 4.

**Fig. 5:**
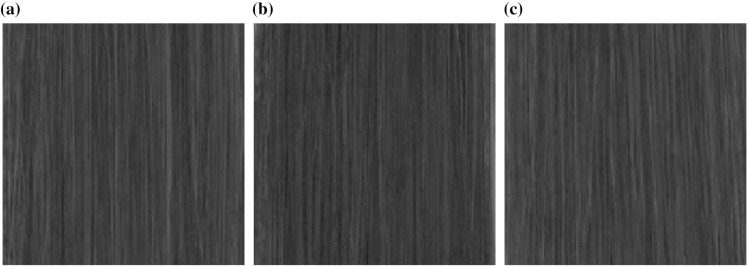
Three different hair swatch colours, A, B and C

**Fig. 6:**
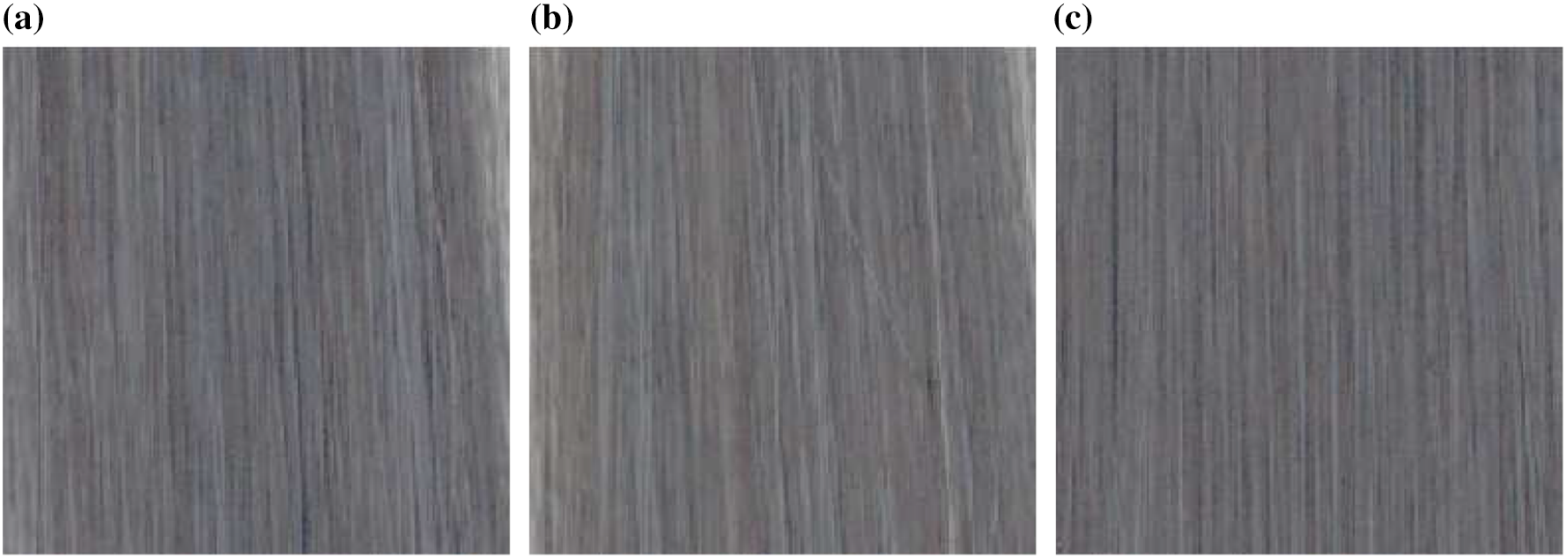
Hair images of three preparation processes applied to a sample of colourant B

#### Hair analysis: different colours

In the first industrial colour texture experiment, each image represents a sample of hair which has undergone one of three different treatments, each of which results in a subtly different colouration of the hair sample. In other words, the swatches we analyse show a change in colour between the images but the *same texture*. More specifically, we wish to classify hair swatches representing three subtly different hair colours denoted A, B and C as shown in Fig. 5 1. We follow the classification approach as outlined in Sect. 4 for each of the four methods, sampling fifty sub-images from the upper half of each image as training data and sampling fifty sub-images from the lower half of each image as the test set. We choose our sub-samples to have dimension 64 x 64.

Table 1 shows all methods have high classification rates. However, of these LS2Wmv achieves the highest correct classification. In this case the physical texture is the same across all images, however the colour changes. Hence, as we would anticipate, the two methods which take coherence into consideration produced the best results, namely the Fourier- and LS2Wmv-based feature classification methods. The VdW method is not competitive, failing to capture all of the structure within the colour textures.

#### Hair analysis: different preparation processes

Our next example analyses images from an experiment in which, after colourant B has been applied to the original image, three different processes are undergone independently (Fig. 6)^2^. In other words, our aim is to distinguish between the three different physical texture processes, where the colour remains the same for each swatch.

Again we follow the classification approach as outlined in Sect. 4. Table 1 shows the classification results for this example, where again the LS2Wmv method achieves the highest classification rate. As may be expected, VdW is reasonably competitive in this case. This is due to the feature vector containing wavelet correlation signatures which take into account the textural change across processes. As all the images are for colourant B they have less variation across the colour planes, so the Fourier approach does not fare well since the images show change in their textural properties.

## Conclusion

In this article we have introduced the multivariate 2D locally stationary wavelet process model and proposed an unbiased and consistent measure of the dependence between two locally stationary channels, namely the locally stationary wavelet coherence. Following this we detailed a full estimation procedure considering important asymptotic properties. We demonstrated the high accuracy of our approach through simulated examples.

We also applied the LS2Wmv modelling approach to a colour texture analysis task, motivated by images encountered in an industrial setting. Both simulated and industrial image examples show that the LS2Wmv has improved performance over other state-of-the-art approaches due to its ability to cope well with both changes in colour and texture features: in this case the coherence contributes to the higher classification rate and thus underlines the importance of coherence in a colour texture setting.

### Electronic supplementary material

Below is the link to the electronic supplementary material.

Supplementary material 1 (zip 8873 KB)

## A Proofs

In this section we provide proofs of the results in Sect. 3 of the article. The proofs are similar in spirit to those in Eckley et al. (2010), but differ in that they extend those results to specifically consider the cross-channel dependence in spectral quantities.

### Proof of Theorem 1.

We first establish uniqueness of the local wavelet cross-spectra of an LS2Wmv process. The structure of the proof is very similar to establishing the uniqueness of auto-spectra in the univariate case considered by Eckley et al. (2010). In order to prove the uniqueness of the multivariate spectral representation, it thus suffices to consider the properties of the cross-spectrum.

Suppose, by way of contradiction, that there exist two cross-spectral representations of the same LS2Wmv process which also possess the same cross-covariance structure. In other words

$$\begin{aligned} c^{p,q}(\mathbf {z},\varvec{\tau }) = \sum _{\eta } S^{(1)(p,q)}_{\eta }(\mathbf {z}) \varPsi _{\eta }(\varvec{\tau }) = \sum _{\eta } S^{(2)(p,q)}_{\eta }(\mathbf {z}) \varPsi _{\eta }(\varvec{\tau }), \end{aligned}$$

where $c^{p,q}(\mathbf {z},\varvec{\tau })$ is defined in Eq.  (3) and $S^{(i)(p,q)}_{\eta }(\mathbf {z}) = W_{\eta }^{p}(\mathbf {z}) W_{\eta }^{q}(\mathbf {z}) \rho _{\eta }^{(p,q)}(\mathbf {z})$ for $i=1,2$ and $p,q \in \{1, \ldots m\} \text{ with } p \ne q$. Let $\varDelta ^{(p,q)}_{\eta }(\mathbf {z}) \equiv S^{(1)(p,q)}_{\eta }(\mathbf {z})-S^{(2)(p,q)}_{\eta }(\mathbf {z})$ be the difference between the two representations. To establish uniqueness we must show that

$$\begin{aligned}&0 = \sum _{\eta } \varDelta ^{(p,q)}_{\eta }(\mathbf {z}) \varPsi _{\eta }(\varvec{\tau }), \quad \forall \ \mathbf {z} \in (0,1)^{2}, \ \forall \ \varvec{\tau } \in \mathbb {Z}^2 \\&\Rightarrow 0 = \varDelta ^{(p,q)}_{\eta }(\mathbf {z}), \quad \forall \ \eta = 1, \ldots , 3J, \ \forall \ \mathbf {z} \in (0,1)^{2}. \end{aligned}$$

What we actually show is the equivalent implication

13 $$\begin{aligned}&0 = \sum _{\eta } \tilde{\varDelta }^{(p,q)}_{\eta }(\mathbf {z}) \varPsi _{\eta }(\varvec{\tau }), \quad \forall \ \mathbf {z} \in (0,1)^{2}, \ \forall \ \varvec{\tau } \in \mathbb {Z}^2 \nonumber \\&\Rightarrow 0 = \tilde{\varDelta }^{(p,q)}_{\eta }(\mathbf {z}), \quad \forall \ \eta = 1, \ldots , 3J, \ \forall \ \mathbf {z} \in (0,1)^{2}, \end{aligned}$$

where $$\tilde{\varDelta }^{(p,q)}_{\eta }(\mathbf {z}) = 2^{-2j(\eta )} \varDelta ^{(p,q)}_{\eta } (\mathbf {z})$$.

Given the assumption, for a given pair of channels *p* and *q*, we have that

14 $$\begin{aligned} 0= & {} \left( \sum _{\eta } \tilde{\varDelta }^{(p,q)}_{\eta }(\mathbf {z}) \varPsi _{\eta }(\varvec{\tau })\right) \left( \sum _{\nu } \tilde{\varDelta }^{(p,q)}_{\nu }(\mathbf {z}) \varPsi _{\nu }(\varvec{\tau })\right) \nonumber \\= & {} \sum _{\varvec{\tau }} \left( \sum _{\eta } \tilde{\varDelta }^{(p,q)}_{\eta }(\mathbf {z}) \varPsi _{\eta }(\varvec{\tau })\right) \left( \sum _{\nu } \tilde{\varDelta }^{(p,q)}_{\nu }(\mathbf {z}) \varPsi _{\nu }(\varvec{\tau })\right) \nonumber \\= & {} \sum _{\eta } \sum _{\nu } \tilde{\varDelta }^{(p,q)}_{\eta }(\mathbf {z}) \tilde{\varDelta }^{(p,q)}_{\nu }(\mathbf {z}) \sum _{\varvec{\tau }} \varPsi _{\eta }(\varvec{\tau }) \varPsi _{\nu } (\varvec{\tau }). \end{aligned}$$

Additionally, using Parseval’s relation, the discrete autocorrelation wavelet inner product matrix can be expressed as

15 $$\begin{aligned} A_{\eta , \nu }= & {} \sum _{\varvec{\tau }} \varPsi _{\eta }(\varvec{\tau }) \varPsi _{\nu } (\varvec{\tau }) \nonumber \\= & {} \left( \frac{1}{2\pi }\right) ^{2} \int {} \int {} \hat{\varPsi }_{\eta }(\varvec{\omega }) \hat{\varPsi }_{\nu }(\varvec{\omega }) d\varvec{\omega }, \end{aligned}$$

where $\hat{\varPsi }_{\eta }(\varvec{\omega }) = |\hat{\psi }_{\eta }(\varvec{\omega })|^{2}$ and $\hat{\varPsi }_{\eta }(\varvec{\omega })$ represents the Fourier transform of $\varPsi$ (Eckley et al. 2010).

Applying Eq. (15) to (14),

16 $$\begin{aligned} 0= & {} \sum _{\eta } \sum _{\varvec{\nu }} \tilde{\varDelta }^{(p,q)}_{\eta }(\mathbf {z}) \tilde{\varDelta }^{(p,q)}_{\nu }(\mathbf {z}) \left( \frac{1}{2\pi }\right) ^{2} \int {} \int {} \hat{\varPsi }_{\eta }(\varvec{\omega }) \hat{\varPsi }_{\nu }(\varvec{\omega }) d\varvec{\omega } \nonumber \\= & {} \int {} \int {} d\varvec{\omega }\left( \sum _{\eta } \tilde{\varDelta }^{(p,q)}_{\eta }(\mathbf {z}) \hat{\varPsi }_{\eta }(\varvec{\omega }) \right) ^{2}. \end{aligned}$$

Since $\sum _{\eta } S_{\eta }^{(p,q)} (\mathbf {z}) < \infty$ uniformly in $\mathbf {z}$, we have $\sum _{\eta }|\varDelta ^{(p,q)}_{\eta }(\mathbf {z})| < \infty$ and hence $\sum _{\eta } 2^{2j(\eta )}|\tilde{\varDelta }^{(p,q)}_{\eta }(\mathbf {z})| < \infty$. For any channel pair (*p*, *q*), $\hat{\varPsi }_{\eta }(\varvec{\omega })$ is continuous on $[-\pi ,\pi ]^{2}$ and in turn $\sum _{\eta } \tilde{\varDelta }^{(p,q)}_{\eta } \hat{\varPsi }_{\eta }(\varvec{\omega })$ is continuous on $[-\pi ,\pi ]^{2}$ as a function of $\varvec{\omega }$. Hence Eq. (16) implies that,

17 $$\begin{aligned} 0 = \sum _{\eta } \tilde{\varDelta }_{\eta }^{(p,q)} (\mathbf {z}) \hat{\varPsi }_{\eta }(\varvec{\omega }), \quad \forall \ \varvec{\omega } \in [-\pi ,\pi ]^{2},\ \forall \ \mathbf {z} \in (0,1)^{2}. \end{aligned}$$

In order to complete this proof we reconsider the Fourier properties of $\varPsi$ as discussed in Eckley et al. (2009). Recall that for $\varvec{\omega }=(\omega _{1},\omega _{2})$,

18 $$\begin{aligned} \left. \begin{array}{lll} \left| \hat{\varPsi }_{j}^{v}(\varvec{\omega })\right| = 2^{2j} \left| m_{1}(2^{j-1}\omega _{1})\right| ^{2} \left| m_{0}(2^{j-1}\omega _{2})\right| ^{2} \\ \quad \times \prod _{s=0}^{j-2} \left| m_{0}(2^{s}\omega _{1}) m_{0}(2^{s}\omega _{2})\right| ^{2} \\ \left| \hat{\varPsi }_{j}^{h}(\varvec{\omega })\right| = 2^{2j} \left| m_{0}(2^{j-1}\omega _{1})\right| ^{2} \left| m_{1}(2^{j-1}\omega _{2})\right| ^{2} \\ \quad \times \prod _{s=0}^{j-2} \left| m_{0}(2^{s}\omega _{1}) m_{0}(2^{s}\omega _{2})\right| ^{2} \\ \left| \hat{\varPsi }_{j}^{d}(\varvec{\omega })\right| = 2^{2j} \left| m_{1}(2^{j-1}\omega _{1})\right| ^{2} \left| m_{1}(2^{j-1}\omega _{2})\right| ^{2} \\ \quad \times \prod _{s=0}^{j-2} \left| m_{0}(2^{s}\omega _{1}) m_{0}(2^{s}\omega _{2})\right| ^{2}. \end{array} \right\} \end{aligned}$$

Let $\tilde{\varDelta }^{(p,q)}_{\eta }:= \tilde{\varDelta }^{(p,q)}_{\eta }(\mathbf {z})$ at some fixed point $\mathbf {z} \in (0,1)^{2}$. Then using Eq. (18) we can rewrite Eq. (17) as follows:

19 $$\begin{aligned} \begin{aligned} 0&= \sum _{\eta =1}^{J} \tilde{\varDelta }_{\eta }^{(p,q)} 2^{2j} \left| m_{1}(2^{j-1}\omega _{1})^{2}\right| \left| m_{0}(2^{j-1}\omega _{2})^{2}\right| \\&\quad \, \times \prod _{s=0}^{j-2} \left| m_{0}(2^{s}\omega _{1}) m_{0}(2^{s}\omega _{2})\right| ^{2} \\&\quad \, + \sum _{\eta =J+1}^{2J} \tilde{\varDelta }_{\eta }^{(p,q)} 2^{2j} \left| m_{0}(2^{j-1}\omega _{1})^{2}\right| \left| m_{1}(2^{j-1}\omega _{2})^{2}\right| \\&\quad \, \times \prod _{s=0}^{j-2} \left| m_{0}(2^{s}\omega _{1}) m_{0}(2^{s}\omega _{2})\right| ^{2} \\&\quad \, + \sum _{\eta =2J+1}^{3J} \tilde{\varDelta }_{\eta }^{(p,q)} 2^{2j} \left| m_{1}(2^{j-1}\omega _{1})^{2}\right| \left| m_{1}(2^{j-1}\omega _{2})^{2}\right| \\&\quad \, \times \prod _{s=0}^{j-2} \left| m_{0}(2^{s}\omega _{1}) m_{0}(2^{s}\omega _{2})\right| ^{2}. \end{aligned} \end{aligned}$$

The RHS of Eq.  (19) is a continuous function of $\varvec{\omega }$ and so must vanish for all $\varvec{\omega } \in [-\pi ,\pi ]^{2}$. In order to show that $\tilde{\varDelta }_{1}$ is zero, we insert $\varvec{\omega }=(\pi ,0)$ into Eq.  (19). This gives,

$$\begin{aligned} 0= & {} \tilde{\varDelta }_{1}^{(p,q)} 4 |m_{1}(\pi )|^{2} |m_{0}(0)|^{2} + \tilde{\varDelta }_{2J+1}^{(p,q)} 4 |m_{1}(\pi )|^{2} |m_{1}(0)|^{2} \nonumber \\= & {} \tilde{\varDelta }_{1}^{(p,q)} 4 \cdot 1 \cdot 1 + \tilde{\varDelta }_{2J+1}^{(p,q)} 4 \cdot 1 \cdot 0, \end{aligned}$$

since $$|m_{1}(\pi )|^{2} $$=1, $$|m_{0}(0)|^{2} $$=1 and therefore $$|m_{1}(2 \pi n)|^{2} $$=0 (Eckley et al. 2009). In other words, we have $$\tilde{\varDelta }_{1}^{(p,q)}=0$$. To show that $$\tilde{\varDelta }_{2J+1}^{(p,q)}=0$$ we reconsider Eq.  (19). Taking $$\varvec{\omega }=(\pi ,\pi )$$ and since $$\tilde{\varDelta }_{1}^{(p,q)}=0$$, we have,

$$\begin{aligned} 0 = \tilde{\varDelta }_{2J+1}^{(p,q)} 4 |m_{1}(\pi )|^{2} |m_{1}(\pi )|^{2} = \tilde{\varDelta }_{2J+1}^{(p,q)} 4 \cdot 1 \cdot 1, \end{aligned}$$

giving $\tilde{\varDelta }_{2J+1}^{(p,q)}=0$. Finally we insert $\varvec{\omega }=(0,\pi )$ into Eq.  (19). Given that $\tilde{\varDelta }_{1}^{(p,q)}=0$ and $\tilde{\varDelta }_{2J+1}^{(p,q)}=0$ we can also derive that $\tilde{\varDelta }_{J+1}^{(p,q)}=0$.

By setting $\varvec{\omega }=(\pi /2,0)$ in Eq.  (19),

$$\begin{aligned} 0= & {} \tilde{\varDelta }_{2}^{(p,q)} 16 |m_{1}(\pi )|^{2} |m_{0}(0)|^{2}+\tilde{\varDelta }_{2J+2}^{(p,q)} 16 |m_{1}(\pi )|^{2} |m_{1}(0)|^{2}\\= & {} \tilde{\varDelta }_{2}^{(p,q)} 16 \cdot 1 \cdot 1 + \tilde{\varDelta }_{2J+2}^{(p,q)} 16 \cdot 1 \cdot 0, \end{aligned}$$

and thus $\tilde{\varDelta }_{2}^{(p,q)}=0$. Proceeding in a similar manner, we can show that $\tilde{\varDelta }_{2J+2}^{(p,q)}=0$ and $\tilde{\varDelta }_{J+2}^{(p,q)}=0$. Continuing with this process recursively setting $\omega = \pi /2^{(j-1)}$ we can show that $\tilde{\varDelta }_{\eta }^{(p,q)}(\mathbf {z})=0, \quad \forall \eta , \forall \mathbf {z} \in (0,1)^{2}$.

As the difference between the two cross-spectra is zero, the cross-spectral representations are uniquely defined given the corresponding LS2Wmv process.

To establish the asymptotic properties of the empirical autocovariance, we first note that

$$\begin{aligned} c_{\mathbf {R}}^{(p,q)}(\mathbf {z},\varvec{\tau })= & {} \text{ Cov }\left( X_{\mathbf {r}}^{(p)},X_{\mathbf {r}+\varvec{\tau }}^{(q)}\right) \\= & {} \mathbb {E}\left( \left( X_{\mathbf {r}}^{(p)} - \mu _{\mathbf {r}}^{(p)}\right) \left( X_{\mathbf {r}+\varvec{\tau }}^{(q)} - \mu _{\mathbf {r}+\varvec{\tau }}^{(q)}\right) \right) . \end{aligned}$$

By the modelling assumptions of LS2Wmv, $\mathbb {E}(X_{\mathbf {r}})=0$ for all $\mathbf {r}$. Hence using Definition 1 $c_{\mathbf {R}}^{(p,q)}(\mathbf {z},\varvec{\tau })$ can be written as

$$\begin{aligned} \sum _{\eta } \sum _{\mathbf {u}} W^{(p)}_{\eta }\left( \frac{\mathbf {u}}{\mathbf {R}}\right) W^{(q)}_{\eta }\left( \frac{\mathbf {u}}{\mathbf {R}}\right) \psi _{\eta ,\mathbf {u-r}} \psi _{\eta ,\mathbf {u-r-\varvec{\tau }}} \mathbb {E}\left( \xi ^{(p)}_{\eta ,\mathbf {u}} \xi ^{(q)}_{\eta ,\mathbf {u}}\right) . \end{aligned}$$

From Eq.  (3) we have,

$$\begin{aligned} \mathbb {E}\left( \xi ^{(p)}_{\eta ,\mathbf {u}} \xi ^{(q)}_{\eta ,\mathbf {u}}\right) = \delta _{\eta ,\eta } \delta _{\mathbf {u}, \mathbf {u}} \rho ^{p,q}_{\eta }\left( \frac{\mathbf {u}}{\mathbf {R}}\right) = \rho ^{p,q}_{\eta }\left( \frac{\mathbf {u}}{\mathbf {R}}\right) . \end{aligned}$$

If $p=q$ then $\rho ^{p,q}_{\eta }(\mathbf {u}/\mathbf {R})=1$ and so the proof follows as in Eckley et al. (2009). For the case where $p \ne q$ we have that $c_{\mathbf {R}}^{(p,q)}(\mathbf {z},\varvec{\tau })$ becomes

$$\begin{aligned}&\sum _{\eta } \sum _{\mathbf {u}} W^{(p)}_{\eta }\left( \frac{\mathbf {u}}{\mathbf {R}}\right) W^{(q)}_{\eta }\left( \frac{\mathbf {u}}{\mathbf {R}}\right) \rho ^{p,q}_{\eta }\left( \frac{\mathbf {u}}{\mathbf {R}}\right) \psi _{\eta ,\mathbf {u-r}} \psi _{\eta ,\mathbf {u-r-\varvec{\tau }}} \\&\quad = \sum _{\eta } \sum _{\mathbf {u}} S^{p,q}_{\eta } \left( \frac{\mathbf {u}}{\mathbf {R}}\right) \psi _{\eta ,\mathbf {u-r}} \psi _{\eta ,\mathbf {u-r-\varvec{\tau }}}. \end{aligned}$$

Finally we consider the absolute difference between the true cross-covariance and the local cross-covariance $|c_{\mathbf {R}}^{(p,q)}(\mathbf {z_{1}},\varvec{\tau }) - c^{(p,q)}(\mathbf {z_{2}},\varvec{\tau })|$. This difference is equal to

$$\begin{aligned}&\left| \sum _{\eta } \sum _{\mathbf {u}} S^{p,q}_{\eta }\left( \frac{\mathbf {u}}{\mathbf {R}}\right) \psi _{\eta ,\mathbf {u-r}} \psi _{\eta ,\mathbf {u-r-\varvec{\tau }}} - \sum _{\eta } S^{p,q}_{\eta }\left( \frac{\mathbf {r}}{\mathbf {R}}\right) \varPsi _{\eta }(\varvec{\tau })\right| \\&\quad = \Big |\sum _{\eta } \sum _{\mathbf {u}} S^{p,q}_{\eta }\left( \frac{\mathbf {u}}{\mathbf {R}}\right) \psi _{\eta ,\mathbf {u-r}} \psi _{\eta ,\mathbf {u-r-\varvec{\tau }}} \\&\quad \quad -\sum _{\eta } S^{p,q}_{\eta }\left( \frac{\mathbf {r}}{\mathbf {R}}\right) \sum _{\mathbf {u}} \psi _{\eta ,\mathbf {u}} \psi _{\eta ,\mathbf {u-\varvec{\tau }}}\Big | \\&\quad = \Big |\sum _{\eta } \sum _{\mathbf {u}} S^{p,q}_{\eta }\left( \frac{\mathbf {u}}{\mathbf {R}}\right) \psi _{\eta ,\mathbf {u-r}} \psi _{\eta ,\mathbf {u-r-\varvec{\tau }}} \\&\quad \quad - \sum _{\eta } S^{p,q}_{\eta }\left( \frac{\mathbf {r}}{\mathbf {R}}\right) \sum _{\mathbf {u}} \psi _{\eta ,\mathbf {u-r}} \psi _{\eta ,\mathbf {u-r-\varvec{\tau }}}\Big |. \end{aligned}$$

Using the Lipschitz continuity of the cross-spectrum $S^{(p,q)}_{\eta }$ Taylor (2013, Lemma 1, Chapter 5), the difference can be bounded by

$$\begin{aligned}&\left| c_{\mathbf {R}}^{(p,q)}(\mathbf {z_{1}},\varvec{\tau }) - c^{(p,q)}(\mathbf {z_{2}},\varvec{\tau })\right| \\&\quad \le \Big |\left( \frac{1}{\min \{R,S\}}\right) \sum _{\eta } \sum _{\mathbf {u}} CB_{\eta }||\mathbf {u-r}|| \psi _{\eta ,\mathbf {u-r}} \psi _{\eta ,\mathbf {u-r-\varvec{\tau }}}\Big |, \end{aligned}$$

and since $\varPsi _{\eta }(\varvec{\tau }) = {\mathcal O}(1)$ uniformly in $\varvec{\tau }$ and the support of $\varPsi _{\eta }(\varvec{\tau })$ is bounded by $K2^{2j(\eta )},$ the distance $||\mathbf {u-r}||$ is also bounded by this amount. Finally we obtain

$$\begin{aligned}&\left| c_{\mathbf {R}}^{(p,q)}(\mathbf {z_{1}},\varvec{\tau }) - c^{(p,q)}(\mathbf {z_{2}},\varvec{\tau })\right| \\&\quad \le \left| \left( \frac{1}{\min \{R,S\}}\right) CK \sum _{\eta } B_{\eta } 2^{2j(\eta )}\right| \\&\quad = {\mathcal O}\left( \frac{1}{\min \{R,S\}}\right) , \end{aligned}$$

since the Lipschitz constants $B_{\eta }$ are uniformly bounded in $\eta$ with $\sum _{\eta } B_{\eta }2^{2j(\eta )} < \infty$ (as stated in Definition 1). $\square$

### Proof of Theorem 2.

Using the LS2Wmv model (Definition 1), the expectation of the local wavelet raw cross-periodogram is given by

$$\begin{aligned} \mathbb {E}(I_{\eta , \mathbf {s}}^{(p,q)})&= \mathbb {E}[(d_{\eta , \mathbf {s}}^{p})(d_{\eta , \mathbf {s}}^{q})] \nonumber \\&= \mathbb {E}\Bigg [\sum _{\mathbf {r_{1}}, \mathbf {r_{2}}} \sum _{\eta _{1},\eta _{2}} \sum _{\mathbf {u_{1}}, \mathbf {u_{2}}} W^{(p)}_{\eta _{1},\mathbf {u_{1}}} \psi _{\eta _{1},\mathbf {u_{1}}}(\mathbf {r_{1}}) \xi ^{(p)}_{\eta _{1},\mathbf {u_{1}}}\\&\quad \, \times W^{(q)}_{\eta _{2},\mathbf {u_{2}}} \psi _{\eta _{2},\mathbf {u_{2}}}(\mathbf {r_{2}}) \xi ^{(q)}_{\eta _{2},\mathbf {u_{2}}} \psi _{\eta ,\mathbf {s}}(\mathbf {r_{1}}) \psi _{\eta ,\mathbf {s}}(\mathbf {r_{2}})\Bigg ]. \end{aligned}$$

Recalling the assumptions on the model innovations (3), the expectation reduces to

$$\begin{aligned} \mathbb {E}(I_{\eta , \mathbf {s}}^{(p,q)})= & {} \sum _{\mathbf {r_{1}}, \mathbf {r_{2}}} \sum _{\eta _{1}} \sum _{\mathbf {u_{1}}} W^{(p)}_{\eta _{1},\mathbf {u_{1}}} W^{(q)}_{\eta _{1},\mathbf {u_{1}}} \rho ^{(p,q)}_{\eta _{1}, \mathbf {u_{1}}} \psi _{\eta _{1},\mathbf {u_{1}}}(\mathbf {r_{1}})\\&\quad \times \psi _{\eta _{1},\mathbf {u_{1}}}(\mathbf {r_{2}}) \psi _{\eta ,\mathbf {s}}(\mathbf {r_{1}}) \psi _{\eta ,\mathbf {s}}(\mathbf {r_{2}}) \\= & {} \sum _{\eta _{1}} \sum _{\mathbf {u_{1}}} S^{(p,q)}_{\eta _{1}, \mathbf {u_{1}}} \sum _{\mathbf {r_{1}}} \psi _{\eta _{1},\mathbf {u_{1}}}(\mathbf {r_{1}}) \psi _{\eta ,\mathbf {s}}(\mathbf {r_{1}})\\&\times \sum _{\mathbf {r_{2}}} \psi _{\eta _{1},\mathbf {u_{1}}}(\mathbf {r_{2}}) \psi _{\eta ,\mathbf {s}}(\mathbf {r_{2}}). \end{aligned}$$

Substituting $\mathbf {u_{1}}=\mathbf {x+s}$ into the equation above we obtain

20 $$\begin{aligned} \mathbb {E}(I_{\eta , \mathbf {s}}^{(p,q)})= & {} \sum _{\eta _{1}} \sum _{\mathbf {x}} S^{(p,q)}_{\eta _{1}, \mathbf {x+s}} \left\{ \sum _{\mathbf {r}} \psi _{\eta _{1}, \mathbf {x+s}}(\mathbf {r}) \psi _{\eta , \mathbf {s}}(\mathbf {r}) \right\} ^{2}\nonumber \\= & {} \sum _{\eta _{1}} \sum _{\mathbf {x}} S^{(p,q)}_{\eta _{1}, \mathbf {x+s}} \Bigg \{\sum _{\mathbf {r}} \psi _{\eta _{1}, \mathbf {x-r}} \psi _{\eta , \mathbf {-r}} \Bigg \}^{2}. \end{aligned}$$

By the assumptions of the model given in Definition 1, $W_{\eta }^{p}$ and $\rho _{\eta }^{p,q}$ are Lipschitz continuous with constants $L^{(p)}_{\eta }$ and $R^{(p,q)}_{\eta }$ respectively. Therefore it follows that the product $S_{\eta }^{p,q}=W_{\eta }^{(p)}W_{\eta }^{(q)}\rho ^{p,q}_{\eta }$ is also Lipschitz continuous (see Taylor (2013, Lemma 1, Chapter 5)). In other words

$$\begin{aligned} \left| S_{\eta }^{(p,q)}\left( \frac{\mathbf {x+s}}{\mathbf {R}}\right) - S_{\eta }^{(p,q)}\left( \frac{\mathbf {s}}{\mathbf {R}}\right) \right| \le \frac{CB^{(p,q)}_{\eta }||\mathbf {x}||}{\min \{R,S\}}, \end{aligned}$$

for Lipschitz constants $CB^{(p,q)}_{\eta }$, with some constant $C \in \mathbb {R}$. Hence

$$\begin{aligned} S_{\eta }^{(p,q)}\left( \frac{\mathbf {x+s}}{\mathbf {R}}\right) = S_{\eta }^{(p,q)}\left( \frac{\mathbf {s}}{\mathbf {R}}\right) + {\mathcal O}\left( \frac{B^{(p,q)}_{\eta }||\mathbf {x}||}{\min \{R,S\}}\right) . \end{aligned}$$

Incorporating this Lipschitz property of the $S^{(p,q)}_{\eta }$ into Eq.  (20), we obtain

$$\begin{aligned} \mathbb {E}(I_{\eta , \mathbf {s}}^{(p,q)})&= \sum _{\eta _{1}} \sum _{\mathbf {x}} S_{\eta _{1}}^{(p,q)}\left( \frac{\mathbf {s}}{\mathbf {R}}\right) \Bigg \{\sum _{\mathbf {r}} \psi _{\eta _{1}, \mathbf {x-r}} \psi _{\eta , \mathbf {-r}} \Bigg \}^{2}\\&\quad + {\mathcal O}\left( \frac{1}{\min \{R,S\}}\right) , \end{aligned}$$

since $\sum _{\eta } B^{(p,q)}_{\eta } < \infty$ and $\left\{ \sum _{\mathbf {r}} \psi _{\eta _{1}, \mathbf {x-r}} \psi _{\eta , \mathbf {-r}}\right\} ^{2}$ is finite. Expanding the square then substituting $\mathbf {r_{0}=r_{2}-r_{1}}$ then gives

$$\begin{aligned} \mathbb {E}(I_{\eta , \mathbf {s}}^{(p,q)})&= \sum _{\eta _{1}} S_{\eta _{1}}^{(p,q)} \left( \frac{\mathbf {s}}{\mathbf {R}}\right) \Bigg \{\sum _{\mathbf {r_{1}}} \sum _{\mathbf {r_{0}}} \psi _{\eta , \mathbf {-r_{1}}} \psi _{\eta , \mathbf {-r_{1}-r_{0}}} \\&\quad \times \sum _{\mathbf {x}} \psi _{\eta _{1}, \mathbf {x-r_{1}}} \psi _{\eta _{1}, \mathbf {x-r_{1}-r_{0}}} \Bigg \}+ {\mathcal O}\left( \frac{1}{\min \{R,S\}}\right) . \end{aligned}$$

Finally we note that each of the summations within the brackets are simply autocorrelation wavelets (see Eckley et al. (2010)). Hence

$$\begin{aligned} \mathbb {E}(I_{\eta , \mathbf {s}}^{(p,q)})= & {} \sum _{\eta _{1}} S_{\eta _{1}}^{(p,q)}\left( \frac{\mathbf {s}}{\mathbf {R}}\right) \sum _{\mathbf {r_{0}}} \varPsi _{\eta _{1}}(\mathbf {r_{0}}) \varPsi _{\eta }(\mathbf {r_{0}}) \\&+\,{\mathcal O}\left( \frac{1}{\min \{R,S\}}\right) \\= & {} \sum _{\eta _{1}} S_{\eta _{1}}^{(p,q)} \left( \frac{\mathbf {s}}{\mathbf {R}}\right) A_{\eta _{1}, \eta } + {\mathcal O}\left( \frac{1}{\min \{R,S\}}\right) , \end{aligned}$$

where *A* is the discrete autocorrelation inner product matrix (see Eckley and Nason (2005) for more details). Therefore by correcting the cross-periodogram with the inverse of the *A* matrix it can be an unbiased estimator of the spectrum.

The result for the variance can be established using a similar strategy, please refer to Taylor (2013) for more details.

### Proof of Theorem 3.

Let *K* be a bounded kernel on $\mathbb {R}^{2}$. In other words, $K:\mathbb {R}^{2} \rightarrow \mathbb {R}$ satisfies $(i)\ \int {}K(\mathbf {x})\ d\mathbf {x} < \infty$ and $(ii)\ |K(\mathbf {x})|< K_{M} < \infty \ \forall \mathbf {x}$. Recall that we use a Nadaraya-Watson kernel estimator to smooth the cross-periodogram. Hence we focus on the asymptotic properties of:

$$\begin{aligned} \mathbb {E}(\tilde{I}^{(p,q)}_{\eta , \mathbf {s}}) = \mathbb {E}\left( \sum _{\mathbf {u}}w_{\mathbf {u}} I^{(p,q)}_{\eta , \mathbf {u}}\right) = \sum _{\mathbf {u}} w_{\mathbf {u}} \mathbb {E}(I^{(p,q)}_{\eta , \mathbf {u}}), \end{aligned}$$

where $w_{\mathbf {u}}=\frac{K_{h}(\mathbf {s-u})}{\sum _{\mathbf {u}}K_{h}(\mathbf {s-u})}$ and define $K_{h}(\mathbf {s-u}) = K(\frac{\mathbf {s-u}}{h})$ for bandwidth $h > 0$. Defining $\lambda = \sum _{\mathbf {u}}K_{h}(\mathbf {s-u})$ and recalling Theorem 2, we have

Setting $\mathbf {u}=\mathbf {s}+\varvec{\tau }$ we obtain

$$\begin{aligned} \mathbb {E}(\tilde{I}^{(p,q)}_{\eta , \mathbf {s}})&= \frac{1}{\lambda } \sum _{\varvec{\tau }} K_{h}(\varvec{\tau })\left[ \sum _{\eta _{1}} S^{(p,q)}_{\eta _{1}}\left( \frac{\mathbf {s}+\varvec{\tau }}{\mathbf {R}}\right) A_{\eta _{1},\eta }\right. \\&\quad \, \left. +\, {\mathcal O}\left( \frac{1}{\min \{R,S\}}\right) \right] . \end{aligned}$$

As discussed in the proof of Theorem 2, $S_{\eta }^{(p,q)}$ is Lipschitz continuous with respect to the $L_{1}$ norm, with constant $CB^{(p,q)}_{\eta }$. Hence the expectation of the smoothed cross-periodogram is

$$\begin{aligned}&\frac{1}{\lambda } \sum _{\varvec{\tau }} K_{h}(\varvec{\tau }) \Bigg [\sum _{\eta _{1}}\left( S^{(p,q)}_{\eta _{1}}\left( \frac{\mathbf {s}}{\mathbf {R}}\right) + {\mathcal O}\left( \frac{B^{(p,q)}_{\eta _{1}}||\varvec{\tau }||}{\min \{R,S\}}\right) \right) A_{\eta _{1},\eta } \\&\qquad + {\mathcal O}\left( \frac{1}{\min \{R,S\}}\right) \Bigg ] \\&\quad = \frac{1}{\lambda } \sum _{\varvec{\tau }} K_{h}(\varvec{\tau })\Bigg [\sum _{\eta _{1}} S^{(p,q)}_{\eta _{1}}\left( \frac{\mathbf {s}}{\mathbf {R}}\right) A_{\eta _{1},\eta } \\&\qquad +\,||\varvec{\tau }|| {\mathcal O}\left( \frac{1}{\min \{R,S\}}\right) + {\mathcal O}\left( \frac{1}{\min \{R,S\}}\right) \Bigg ], \end{aligned}$$

where $A_{\eta _{1},\eta }= {\mathcal O}(2^{2j(\eta _{1})})$ and since $\sum _{\eta _{1}} B_{\eta _{1}}^{(p,q)} 2^{2j(\eta _{1})} < \infty .$ Therefore,

21 $$\begin{aligned} \mathbb {E}(\tilde{I}^{(p,q)}_{\eta , \mathbf {s}})= & {} \frac{1}{\lambda } \sum _{\varvec{\tau }} K_{h}(\varvec{\tau })\Big ( \sum _{\eta _{1}} S^{(p,q)}_{\eta _{1}}\left( \frac{\mathbf {s}}{\mathbf {R}}\right) A_{\eta _{1},\eta }\Big ) \nonumber \\&+\frac{1}{\lambda } \sum _{\varvec{\tau }} K_{h}(\varvec{\tau })\bigg ((||\varvec{\tau }||+1) {\mathcal O}\left( \frac{1}{\min \{R,S\}}\right) \bigg ) \nonumber \\= & {} \tilde{I}_{1} + \tilde{I}_{2}, \end{aligned}$$

where $\tilde{I}_{1}= \frac{1}{\lambda } \sum _{\varvec{\tau }} K_{h}(\varvec{\tau })\Big ( \sum _{\eta _{1}} S^{(p,q)}_{\eta _{1}}\left( \frac{\mathbf {s}}{\mathbf {R}}\right) A_{\eta _{1},\eta }\Big )$ and $\tilde{I}_{2} = \frac{1}{\lambda } \sum _{\varvec{\tau }} K_{h}(\varvec{\tau })\bigg ((||\varvec{\tau }||+1) {\mathcal O}\left( \frac{1}{\min \{R,S\}}\right) \bigg )$. First we focus on $\tilde{I}_{1}$:

$$\begin{aligned} \tilde{I}_{1}= & {} \frac{1}{\lambda } \sum _{\varvec{\tau }} K_{h}(\varvec{\tau })\Big ( \sum _{\eta _{1}} S^{(p,q)}_{\eta _{1}}\left( \frac{\mathbf {s}}{\mathbf {R}}\right) A_{\eta _{1},\eta }\Big ) \\= & {} \sum _{\eta _{1}} S^{(p,q)}_{\eta _{1}}\left( \frac{\mathbf {s}}{\mathbf {R}}\right) A_{\eta _{1},\eta }, \end{aligned}$$

since $\sum _{\mathbf {u}} w_{\mathbf {u}} = \frac{1}{\lambda } \sum _{\varvec{\tau }} K_{h}(\varvec{\tau })=1$. In order to evaluate the second term in Eq.  (21), we consider the number of lattice points, $(2 \lfloor h \rfloor + 1)^{2}$, in the support of a kernel of bandwidth *h*. More specifically we divide both the numerator and denominator by this quantity. In so doing we obtain an expression for $\tilde{I}_{2}$ as:

$$\begin{aligned} \frac{ \Bigg (\frac{1}{(2 \lfloor h \rfloor + 1)^{2}} \sum _{\varvec{\tau }} K_{h}(\varvec{\tau }) \Bigg [(||\varvec{\tau }||+1) {\mathcal O}\left( \frac{1}{\min \{R,S\}}\right) \Bigg ]\Bigg )}{ \Bigg (\frac{\lambda }{(2 \lfloor h \rfloor + 1)^{2}}\Bigg )}. \end{aligned}$$

We now analyse the numerator and denominator of $\tilde{I}_{2}$ separately, denoting these by $\tilde{I}_{2,N}$ and $\tilde{I}_{2,D}$ respectively. The numerator is given by,

$$\begin{aligned} \tilde{I}_{2,N}<&\frac{\sum _{\varvec{||\tau ||}_{1}< \lfloor h \rfloor } K_{h}(\varvec{\tau })\Big ((\lfloor h \rfloor +1) {\mathcal O}\left( \frac{1}{\min \{R,S\}}\right) \Big )}{(2 \lfloor h \rfloor + 1)^{2}} \\= & {} \frac{\Big (( \lfloor h \rfloor +1) {\mathcal O}\left( \frac{1}{\min \{R,S\}}\right) \Big )}{(2 \lfloor h \rfloor + 1)^{2}} \\= & {} \left( \frac{1}{2 \lfloor h \rfloor +1}\right) {\mathcal O}\left( \frac{1}{\min \{R,S\}}\right) , \end{aligned}$$

since

$$\begin{aligned} 0< \sum _{||\varvec{\tau }||_{1}< \lfloor h \rfloor } K_{h} (\varvec{\tau })< \int _{||\varvec{x}||_{2}<h}{K_{h}(\varvec{x})d\varvec{x}}=1, \end{aligned}$$

and $||\varvec{\tau }||_{1}=|\tau _{1}|+|\tau _{2}| < \lfloor h \rfloor$. The denominator of $\tilde{I}_{2}$ is given by $\tilde{I}_{2,D} = \frac{\lambda }{(2 \lfloor h \rfloor + 1)^{2}}$, where $\lambda = \sum _{\varvec{\tau }}K_{h}(\varvec{\tau })$. We have,

$$\begin{aligned} \tilde{I}_{2,D} < \frac{1}{(2 \lfloor h \rfloor + 1)^{2}} \times (2 \lfloor h \rfloor + 1)^{2} K_{M} = {\mathcal O}(1), \end{aligned}$$

since $K(x) \le K_{M} < \infty$ where $K_{M}$ is the maximum point of the kernel. Combining the two terms of (21), the expectation of the smoothed cross-periodogram $\mathbb {E}(\tilde{I}^{(p,q)}_{\eta , \mathbf {s}})$ is thus

$$\begin{aligned} \sum _{\eta _{1}} S^{(p,q)}_{\eta _{1}}\left( \frac{\mathbf {s}}{\mathbf {R}}\right) A_{\eta _{1},\eta } + \left( \frac{1}{2\lfloor h\rfloor +1}\right) {\mathcal O}\left( \frac{1}{\min \{R,S\}}\right) . \end{aligned}$$

$$\square $$

## Footnotes

1 Note that these images have been artificially lightened for display purposes.

2 Note that these images have been artificially lightened for display purposes.
